# Ram Vinay Pandey und Nadine Ballicas erhalten den Heinrich Auspitz Preis der ÖGDV 2023

**DOI:** 10.1111/ddg.15618_g

**Published:** 2025-01-08

**Authors:** Nadine Ballicas, Ram Vinay Pandey

Die ÖGDV ehrt mit dem Heinrich Auspitz Preis jedes Jahr hervorragende wissenschaftliche Arbeiten aus dem Gebiet der Immundermatologie und entzündlichen Dermatosen. Wir gratulieren den Preisträger:innen Ram Vinay Pandey und Nadine Ballicas, die sich im Folgenden mit ihren prämierten Arbeiten vorstellen.

Die allogene hämatopoetische Stammzelltransplantation (allo‐HSCT) ist eine wirksame Form der Tumorimmuntherapie, mit weltweit steigenden Zahlen von Transplantationen. Eine wesentliche Einschränkung der allo‐HSCT ist jedoch die akute Spender‐gegen Empfänger‐Reaktion (engl. graft‐versus‐host disease; GVHD), ein Zustand, bei dem Spender‐T‐Zellen Wirtsgewebe angreifen und mehrere Organe, insbesondere Haut, Leber und Magen‐Darm‐Trakt, beeinträchtigen. Trotz Standardprophylaxe entwickelt sich die GVHD bei etwa 50% der Empfänger und ist eine häufige Todesursache. Das Ansprechen auf die systematische Therapie mit Steroiden reicht von 60% bei Patienten mit milder GVHD, bis zu lediglich 30–40% bei Patienten mit einer schweren Erkrankung.^1^ In dieser Studie wurden mittels bulk‐RNA‐ und ATAC‐Sequenzierung epigenetische Regulatoren von T‐Zellen im Blut und in der Haut von Patienten nach Stammzelltransplantation untersucht. Es zeigte sich, dass vor allem epigenetische Regulatoren wie der BRG1/BRM‐assoziierte Faktor‐Chromatin‐Remodelling‐Komplex und Histon‐Deacetylasen (HDACs) an der Regeneration der T‐Zellpopulationen beteiligt sind. Mittels Klasse‐I‐HDAC‐Inhibitoren konnte eine Modulation von verschiedenen T‐Zellpopulationen beobachtet werden. Insbesondere am 14. Tag nach HSCT wurde die höchste Anzahl offener Chromatinregionen in kutanen T‐Zellen beobachtet. Dies deutet auf eine stärkere Umgestaltung des Chromatins hin, welche durch eine weitere Differenzierung von Spender‐T‐Zellen durch TCR‐Stimulation in der Haut des Empfängers ausgelöst wird.^2^ Zusammenfassend unterstreicht diese Studie den Nutzen von Multi‐Omics‐Analysen und die Bedeutung der epigenetischen Regulierung bei der Wiederherstellung von T‐Zellen nach HSCT.^3^

Der Artikel “Disturbances in microbial skin recolonization and cutaneous immune response following allogeneic stem cell transfer” untersucht die Auswirkungen der allogenen Stammzelltransplantation auf die Zusammensetzung des Hautmikrobioms, insbesondere beim Auftreten der Spender‐gegen‐Empfänger‐Reaktion (engl.:“Graft‐versus‐Host‐Disease”, “GvHD”). Rund 50% aller Empfänger:innen einer Stammzellspende sind von dieser Erkrankung betroffen, welche fast immer die Haut betrifft.^4^ Die Studie zeigt, dass die Bakterienvielfalt auf der Haut beim Auftreten einer GvHD abnimmt, vor allem bei Patient:innen mit einer schweren Form der Erkrankung. Zudem kommt es zu einer Vermehrung von Staphylokokken spp. im Bereich der GvHD Läsionen. Besonders hervorzuheben ist, dass sich bereits eine Reduktion der Bakterienvielfalt nachweisen lässt, bevor noch die ersten Symptome auftreten. Auf diese Weise könnten schwere Verläufe der Haut GvHD in Zukunft bereits früher erkannt und behandelt werden. Darüber hinaus geht die Arbeit auf die Auswirkungen der Transplantation auf das kutane Immunsystem ein, welches mittels einer speziell entwickelten Bildanalyse von gefärbten Hautschnitten untersucht wurde.^5^ Dabei wurde eine vermehrte Nähe Staphylokokken und CD45+ Leukozyten in der Haut von GvHD Patienten beobachtet, die mit einer Hochregulierung von Genen, welche für die Antigen‐Präsentation notwendig sind, einhergingen. Schlussendlich konnten wir mit den Daten zeigen, dass das Hautmikrobiom eine bisher noch unbekannte Rolle bei der Haut GvHD spielt. Weitere Forschungen in diesem Bereich könnten zur Entwicklung gezielter Interventionen oder Therapien führen, die darauf abzielen, das mikrobielle Gleichgewicht der Haut wiederherzustellen sowie die Immunabwehr nach der Transplantation zu stärken.
